# Frailty and bone health in European men

**DOI:** 10.1093/ageing/afw205

**Published:** 2016-11-16

**Authors:** Michael J. Cook, Alexander Oldroyd, Stephen R. Pye, Kate A. Ward, Evelien Gielen, Rathi Ravindrarajah, Judith E. Adams, David M. Lee, Gyorgy Bartfai, Steven Boonen, Felipe Casanueva, Gianni Forti, Aleksander Giwercman, Thang S. Han, Ilpo T. Huhtaniemi, Krzysztof Kula, Michael E. Lean, Neil Pendleton, Margus Punab, Dirk Vanderschueren, Frederick C. Wu, Terence W. O'Neill, for the EMAS Study Group

**Affiliations:** 1 Arthritis Research UK Centre for Epidemiology, Division of Musculoskeletal & Dermatological Sciences, School of Biological Sciences, Faculty of Biology, Medicine and Health, Manchester Academic Health Science Centre, The University of Manchester, Manchester, UK; 2 NIHR Manchester Musculoskeletal Biomedical Research Unit, Central Manchester NHS Foundation Trust, Manchester Academic Health Sciences Centre, Manchester, UK; 3 MRC Human Nutrition Research, Elsie Widdowson Laboratory, Cambridge, UK; 4 Gerontology and Geriatrics, Department of Clinical and Experimental Medicine, KU Leuven, Leuven, Belgium; 5 Primary Care & Public Health Services, King's College London, London, UK; 6 Radiology and Manchester Academic Health Science Centre, The Royal Infirmary, The University of Manchester, Manchester, UK; 7 Cathie Marsh Institute for Social Research, School of Social Sciences, The University of Manchester, ManchesterM13 9PL, UK; 8 Department of Obstetrics, Gynaecology and Andrology, Albert Szent-György Medical University, Szeged, Hungary; 9 University Division of Geriatric Medicine, Katholieke Universiteit Leuven, Leuven, Belgium; 10 Department of Medicine, Santiago de Compostela University, Complejo Hospitalario Universitario de Santiago (CHUS), CIBER de Fisiopatologia Obesidad y Nutricion (CIBERobn), Instituto Salud Carlos III,Santiago de Compostela, Spain; 11 Endocrine Unit, Department of Clinical Physiopathology, University of Florence, Florence, Italy; 12 Scanian Andrology Centre, Department of Urology, Malmö University Hospital, University of Lund, Sweden; 13 Egham & Department of Endocrinology, Ashford and St Peter's NHS Foundation Trust, Institute of Cardiovascular Research, Royal Holloway, University of London (ICR2UL), Chertsey, UK; 14 Department of Reproductive Biology, Imperial College London, London, UK; 15 Department of Andrology and Reproductive Endocrinology, Medical University of Lodz, Poland; 16 Human Nutrition, University of Glasgow, Glasgow, UK; 17 Division of Neuroscience and Experimental Psychology, University of Manchester, Salford Royal Hospital, Salford, UK; 18 Andrology Unit, United Laboratories of Tartu University Clinics, Tartu, Estonia; 19 Department of Andrology and Endocrinology, Katholieke Universiteit Leuven, Leuven, Belgium; 20 Andrology Research Unit, Centre for Endocrinology and Diabetes, University of Manchester, Manchester, UK

**Keywords:** Frailty, male health, heel ultrasound, bone mineral density, older people

## Abstract

**Background:**

frailty is associated with an increased risk of fragility fractures. Less is known, however, about the association between frailty and bone health.

**Methods:**

men aged 40–79 years were recruited from population registers in eight European centres for participation in the European Male Aging Study. Subjects completed a comprehensive assessment which included quantitative ultrasound (QUS) scan of the heel (Hologic-SAHARA) and in two centres, dual-energy bone densitometry (dual-energy x-ray absorptiometry, DXA). Frailty was defined based on an adaptation of Fried's phenotype criteria and a frailty index (FI) was constructed. The association between frailty and the QUS and DXA parameters was determined using linear regression, with adjustments for age, body mass index and centre.

**Results:**

in total, 3,231 subjects contributed data to the analysis. Using the Fried categorisation of frailty, pre-frail and frail men had significantly lower speed of sound (SOS), broadband ultrasound attenuation (BUA) and quantitative ultrasound index (QUI) compared to robust men (*P<* 0.05). Similar results were seen using the FI after categorisation into ‘high’, ‘medium’ and ‘low’ levels of frailty. Using the Fried categorisation, frail men had lower femoral neck bone mineral density (BMD) compared to robust men (*P* < 0.05), but not lower lumbar spine BMD. Using the FI categorisation, a ‘high’ level of frailty (FI > 0.35) was associated with lower lumbar spine BMD (*P* < 0.05) when compared to those with low (FI < 0.2), but not lower femoral neck BMD. When analysed as a continuous variable, higher FI was linked with lower SOS, BUA and QUI (*P* < 0.05).

**Conclusions:**

optimisation of bone health as well as prevention of falls should be considered as strategies to reduce fractures in frail older people.

## Background

The aging process is characterised by a complex alteration of anatomical, physiological and psychological factors. In a significant number of individuals, these changes can result in frailty, a syndrome that has been defined as ‘an excess vulnerability to stressors, with reduced ability to maintain homoeostasis after a destabilising event’ [1]. Frailty is linked with adverse health outcomes including an increased risk of falls and institutionalisation [2]. Frailty has also been linked in prospective studies with an increased risk of future fractures, though whether this is related to the increased susceptibility to falls or whether there is in addition an associated reduction in bone strength remains uncertain [3]. Previous studies have investigated the relationship between frailty and bone mineral density (BMD) [1, 4–11], however, the results have been somewhat discrepant. Some, though not all, suggest an association between frailty and markers of bone strength, including calcaneal BMD [4] and femoral neck or lumbar spine BMD [5, 6, 8]. However, there are few data in men. Such data are important; knowledge of the factors which predispose to fracture, including bone strength, may help improve targeted preventative measures in this high risk group. If frailty is linked with reduced bone strength, then fracture prevention measures should include not just falls prevention but also measures to optimise bone strength. The aim of this study was to investigate the relationship between frailty and bone health defined using both BMD and quantitative ultrasound (QUS) measurements, in a population of community dwelling European men.

## Methods

### Participants

Subjects were recruited for participation in the European Male Aging Study (EMAS) from eight European centres (Florence, Italy; Leuven, Belgium; Malmö, Sweden; Manchester, UK; Santiago de Compostela, Spain; Łódź, Poland; Szeged, Hungary; Tartu, Estonia). Participants completed a postal questionnaire and attended a research centre for further assessment. Ethical approval for the study was obtained in accordance with local institutional requirements in each centre. Each participant provided written consent.

### Assessments

The postal questionnaire included items concerning health and lifestyle [12]. Participants were asked also whether they were currently receiving treatment for a range of medical conditions. The interviewer assisted questionnaire included the short form (SF36), the Physical Activity Scale for the Elderly [13], Reuben's Physical Performance test [14], Beck's Depression Inventory [15] and the Tinetti balance and postural stability index [16]. A range of anthropometric measurements were performed including mid-upper arm circumference (cm) and triceps skinfold thickness (mm). Body mass index (BMI) was calculated as weight (kg) divided by the square of height (m).

### QUS of the heel

QUS of the left heel was performed in all subjects with the Sahara Clinical Sonometer (Hologic, Inc, Bedford, MA, USA) using a standardised protocol in all centres. Outputs included broadband ultrasound attenuation (BUA, dB/MHz), speed of sound (SOS, m/s) and quantitative ultrasound index (QUI) which is a parameter derived from SOS and BUA (0.41*SOS+0.41*BUA-571). Short term precision was measured by performing duplicate measurements in 20 randomly selected subjects from one centre (Leuven, Belgium). The *in vivo* coefficients of variation (CVs) were 2.8% and 0.3% for BUA and SOS, respectively. Repeat measurements (*n* = 10) were performed on a roving phantom at each of the eight centres. Standardised CVs (SCVs) for within machine variability ranged by centre: for SOS, from 1.0% to 5.6%, and BUA from 0.7% to 2.7%. SCVs for between machine variability were 4.8% for BUA and 9.7% for SOS [17].

### Dual-energy x-ray absorptiometry

Areal bone mineral density (BMDa) scans were carried out in the Manchester and Leuven subsets of EMAS (*N* = 735). Both sites used dual-energy x-ray absorptiometry (DXA) QDR 4500A devices from the same manufacturer (Hologic, Inc, Waltham, MA, USA). BMDa was measured at the lumbar spine (L1–L4) and proximal femur (total region). The precision errors in Leuven were 0.57% and 1.28% at the lumbar spine and total femur region, respectively. In Manchester, these precision errors were 0.97% and 2.04% at the lumbar spine (L1–L4) and proximal femur (total region), respectively. Both devices were cross-calibrated with the European Spine Phantom [18].

### Frailty

Frailty status was determined using a phenotypic definition adapted from Fried and colleagues based on five criteria: sarcopenia, exhaustion, slowness, weakness and low activity. Details of the EMAS frailty phenotype (FP) criteria are reported elsewhere [19]. Briefly, ‘sarcopenia’ was based on mid-upper arm muscle circumference (mid-upper arm circumference – 3.14 × triceps skinfold thickness), the threshold being the lowest 10% from men over 65 years. ‘Exhaustion’ was defined using BDI-II energy and fatigue items, responses being ‘I don't have enough energy to do very much/do anything’ or ‘I am too tired or fatigued to do a lot of/most of the things I used to’, respectively. ‘Slowness’ from the PPT 50 foot walk test, the threshold being the slowest 20% stratified by height for men 65 years and older. ‘Weakness’ from the Tinetti 5 chair stand test, the threshold being the slowest 10% for those 65 years and over, or who were unable to complete the test, and ‘low activity’ from the PASE score, the threshold being the lowest 20% for 65+ years. The Fried frailty category variable was constructed as follows: 0 criteria = robust (not frail), 1 or 2 criteria as pre-frail and those with 3 or more criteria as frail. Men with missing data for one or more components of the Fried criteria were not included in the analysis. We also calculated a frailty index (FI). An FI represents the number of defined health deficits present in an individual divided by the number of health deficits considered [20, 21]. In EMAS, 39 potential deficits were evaluated and included in the FI. These represent symptoms, signs or functional impairments that accumulate with age and are individually related to adverse outcomes. Details of the EMAS FI are reported elsewhere [22]. We analysed the FI both as a continuous variable and as a categorical variable using the threshold levels suggested by Kulminski and colleagues: low (robust), FI ≤ 0.2; medium (pre-frail), 0.2 < FI ≤ 0.35; and high (frail), FI > 0.35 [23]. Men with missing data for eight components or more (20%) of the FI were not included in the analysis.

### Statistical analysis

Descriptive statistics were used to summarise subject characteristics. Linear regression analysis was used to determine the association between frailty category (using the phenotype and FI definitions), and ultrasound and DXA bone parameters, adjusted for age, centre and BMI with the results expressed as β-coefficients and 95% confidence intervals (CIs). In these analyses the β-coefficients represent the absolute difference in bone parameter among a particular frailty group compared to the referent value (either the low or robust category). From the adjusted linear regression models, post estimation of the marginal mean values of bone parameters for each frailty category was performed. We looked also at the relationship between the component features which make up the FP criteria and bone parameters. We looked also at the association between the FI expressed as a continuous variable and the bone parameters; in this analysis the β-coefficients represent the change in FI for each unit change in bone parameter. All statistical analyses were performed using STATA version 11.2 (http://www.stata.com).

## Results

### Subject characteristics

A total of 3,369 men were recruited to EMAS. Of these participants, 29 men were excluded because they were taking bone active therapies (calcium, vitamin D, bisphosphonates, glucocorticoids). A further 109 participants who did not have QUS of the heel measured at baseline were also excluded, leaving 3,231 men for analysis. Mean (SD) age of these subjects was 59.9 (11.0) years and mean (SD) BMI was 27.6 (4.0) kg/m^2^. In total, 735 men from the Manchester and Leuven centres had BMD measurements performed. Subject characteristics are shown in Table 1. Based on the Fried definition of frailty, 783 (26.4%) of the 2,965 men in whom it was possible to characterise frailty were defined as pre-frail and 72 (2.4%) as frail. The proportion of men who were frail increased with age from 0.1% at age 40–49 years to 6.9% at age 70–79 years. The proportion of men satisfying each of the component criteria varied from 5.7% (sarcopenia) to 10.3% (low physical activity). The median FI was 0.09 (IQR 0.04, 0.15). Of the 735 who also underwent BMD measurement, 151 (20.5%) were pre-frail and 11 (1.5%) frail using the Fried FP.

**Table 1.** Subject characteristics

Variable		Statistic
*Heel quantitative ultrasound*	*N* = 3231	*Mean *(*SD*)
BUA (dB/MHz)		80.3 (18.9)
SOS (m/s)		1550.9 (34.1)
QUI		97.8 (21.2)
*Areal bone mineral density*	*N* = 735	*Mean *(*SD*)
Lumbar spine (g/cm^2^)		1.055 (0.175)
Femoral neck (g/cm^2^)		0.807 (0.127)
*Frailty index*	*N* = 2450	*n *(*%*)
Low		2016 (82.3)
Medium		342 (14.0)
High		92 (3.8)
*Fried frailty phenotype*	*N* = 2965	*n *(*%*)
Robust		2110 (71.2)
Pre-frail		783 (26.4)
Frail		72 (2.4)
*Components of Fried frailty phenotype*	*N* = 2965	*n *(*%*)
Low physical activity		305 (10.3)
Exhaustion		239 (8.1)
Slowness		291 (9.8)
Weakness		198 (6.7)
Sarcopenia		168 (5.7)

### FP and bone health

**Figure 1.**
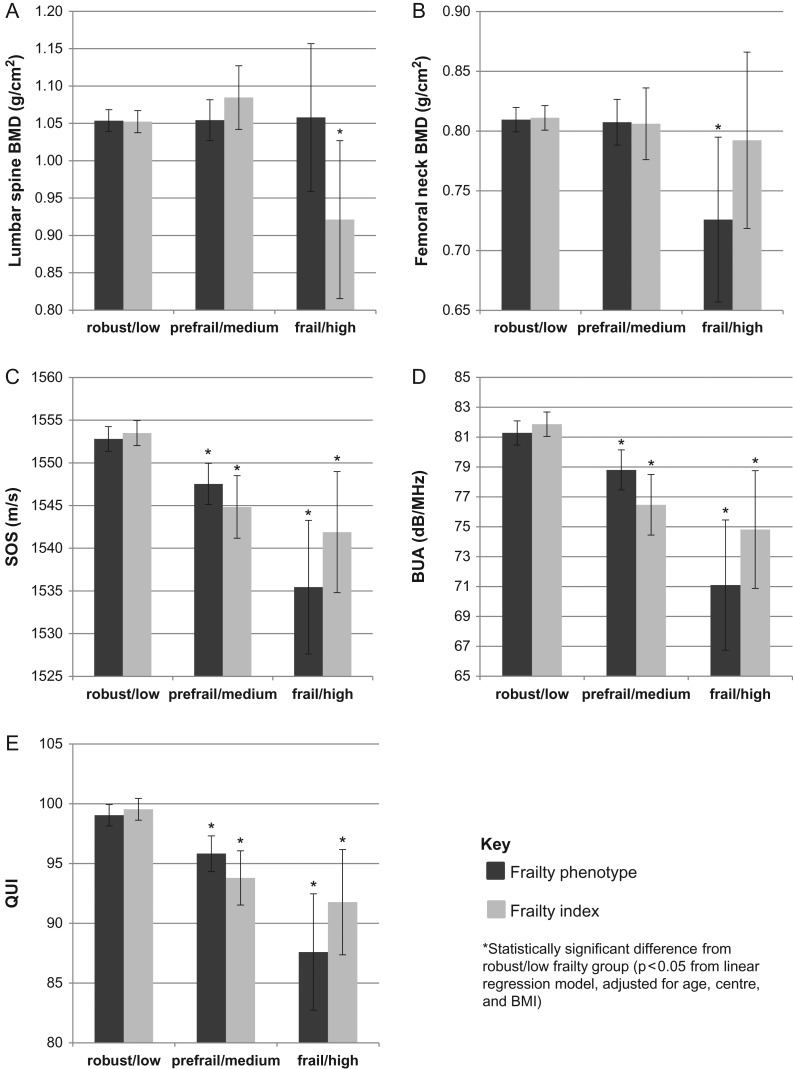
Marginal mean values (95% CI) of QUS and DXA parameters by FP and FI category

After adjustment, and compared to those who were robust, frailty defined using the phenotype approach, was associated with a reduced SOS (β coefficient −17.4; 95% CI −25.4, −9.4), BUA (β coefficient −10.2; 95% CI −14.6,-5.7) and QUI (β coefficient −11.4; 95% CI −16.4, −6.5), see Table 2 and also Figure 1. Pre-frailty was linked also significantly with reduced heel ultrasound parameters though the β coefficients were smaller. There was no association between frailty and lumbar spine BMD, though frailty was associated with reduced femoral neck BMD (β coefficient −0.084; 95% CI −0.15, −0.014). Each individual component of the FP was linked with a lower SOS, and this was statistically significant in the adjusted model for low physical activity, exhaustion, slow walking speed and weakness, see Supplementary Table S1, available at *Age and Ageing* online. The results were broadly similar for BUA, apart from low physical activity (QUI and BUA) and weakness (BUA) which were not statistically significant. For femoral neck BMD, low physical activity, exhaustion, weakness and sarcopenia were linked with lower BMD though this was statistically significant for exhaustion only, see Supplementary Table S1, available at *Age and Ageing* online.

**Table 2.** Frailty and bone health parameters

Variable	Heel quantitative ultrasound			Areal bone mineral density	
	Adjusted β coefficient (95% CI)^a^			Adjusted β coefficient (95% CI)^a^	
	SOS (m/s)	BUA (dB/MHz)	QUI	Lumbar spine	Femoral neck
*Fried frailty category*	*n* = 2961	*n* = 2961	*n* = 2961	*n* = 673	*n* = 671
Robust	Referent	Referent	Referent	Referent	Referent
Pre-frail	−5.3 (−8.1, −2.4)***	−2.5 (−4.1, −0.9)**	−3.2 (−5.0, −1.4)***	0.00062 (−0.031, 0.032)	−0.0021 (−0.024, 0.020)
Frail	−17.4 (−25.4, −9.4)***	−10.2 (−14.6, −5.7)***	−11.4 (−16.4, −6.5)***	0.0044 (−0.096, 0.10)	−0.084 (−0.15, −0.014)*
*Frailty index category*	*n* = 2442	*n* = 2442	*n* = 2442	*n* = 581	*n* = 580
Low	Referent	Referent	Referent	Referent	Referent
Medium	−8.7 (−12.6, −4.7)***	−5.4 (−7.6, −3.2)***	−5.7 (−8.2, −3.3)***	0.032 (−0.014, 0.078)	−0.0049 (−0.037, 0.027)
High	−11.6 (−18.9, −4.3)**	−7.0 (−11.1, −3.0)*	−7.8 (−12.3, −3.2)**	−0.13 (−0.24, −0.024)*	−0.019 (−0.0034, −0.0015)
Frialty index^b^	−44.8 (−59.5, −30.0)***	−25.5 (−33.7, −17.3)***	−28.7 (−37.9, −19.6)***	−0.027 (−0.21, 0.16)	−0.083 (−0.21, 0.044)

Lumbar spine and femoral neck BMD is measured in g/cm^2^.*P < 0.05; **P < 0.01; ***P < 0.001.

^a^Adjusted for age, BMI and centre. Results are presented as adjusted β coefficients with robust/low frailty category as the referent group. These results represent the expected difference in bone parameters for each group, compared to the robust category.

^b^FI expressed as a continuous measure.

### FI and bone health

An increase in FI, assessed as a continuous measure, was significantly associated with lower BUA, SOS and QUI in the adjusted model, (β coefficient −25.5; 95% CI −33.7, −17.3, β coefficient −44.8; 95% CI −59.5, −30.0, and β coefficient −28.7; 95% CI −37.9, −19.6, respectively), see Figure 1 and Table 2. Compared to those with low FI, those who were categorised as high FI (FI > 0.35) had significantly lower SOS (β coefficient = −11.6; 95% CI −18.9, −4.3), BUA (β coefficient −7.0; 95% CI −11.1, −3.0), QUI (β coefficient −7.8; 95% CI −12.3, −3.2) and lumbar spine BMD (β coefficient −0.13; 95% CI −0.24, −0.024).

## Discussion

In this population survey we found a significant association between frailty and bone health parameters including low BUA, SOS, QUI and also femoral neck BMD using the Fried categorisation and lumbar spine BMD using the FI categorisation, though the magnitude of these effects was relatively small. There was some evidence of a dose-response effect with those classified as pre-frail having BUA, SOS and QUI levels intermediate between those who were robust and those who were frail. All the component phenotype criteria were associated with reduced BUA and/or SOS/QUI though the effect appeared to be more marked for the slow walking speed and exhaustion criteria.

Epidemiological studies in men and women have shown that frailty is linked with an increased risk of falls and future fractures [19, 24–27]. There are, however, surprisingly few studies which have looked at the link between bone health and frailty, particularly in men. To our knowledge there are no data looking at the association between frailty and QUS parameters. Most cross-sectional studies have shown, after adjustment for age, no association between frailty and calcaneal, lumbar spine or femoral neck BMD [6, 8]. One study of older (>65 years) community-dwelling participants (82% female) found that frailty, defined using a modified Vulnerable Elders Survey (VES-13), was associated with lower BMD of the calcaneus, though the prevalence of frailty was higher than in other comparative studies at 44% [4]. A prospective study of community-dwelling men aged 70–97 years found no association between baseline frailty, defined by the Fried FP, and total hip BMD over a mean follow-up of 2.2 years after adjusting for age [5]. In contrast, a study of 235 community-dwelling women aged >70 years, found that frailty at baseline, defined by the VES-13, predicted lower total hip and lumbar spine BMD 1 year later, although no association was seen between baseline frailty and baseline total hip or lumbar spine BMD [9]. The inconsistency in the literature could, in part, be explained by differences in study design, sex, age and ethnicity of participants and choice of frailty instrument. Our data are consistent with an association between frailty and bone health parameters. The mechanism linking frailty and bone health is likely to be multifactorial and include a reduction in muscle mass and strength, reduced loading due to immobility, a decline in sex hormones, impaired nutrition including protein intake, the presence of chronic disease and also dysregulated inflammation [28].

Our study has a number of strengths; it was large, population based, and used standard methods in both conduct and assessment. There are though a number of limitations which need to be considered in interpreting the analysis. The response rate for participation 41%, with those who declined to take part being older, more likely to be current smokers and reporting experiencing less pain lasting at least one day in the past month, than those who participated [12]. It seems unlikely, however that any such selection factors would impact on the findings reported, which were based on internal comparison of those who took part. The Fried phenotype definition of frailty developed in EMAS was adapted from the original definition, utilising the data available and instruments used in EMAS. It has though been shown to be associated with falls, impaired quality of life [19], and mortality [29]. Among those with BMD measurements performed (*N =* 735) the prevalence of frailty was lower than those who did not have the measurements performed (1.5% versus 2.4%). However it seems unlikely that this would have influenced findings concerning the association between BMD and frailty which was based on an internal comparison of those in whom measurements were performed.

Men with missing data for one or more components of the Fried FP were not included in the analysis; again it seems unlikely that this would have had an influence on our findings relating to the association between frailty and bone parameters. Our study was cross-sectional and therefore it is not possible to determine the temporal nature of the observed associations. It seems unlikely though that reduced bone density per se would lead to an increase in the risk of frailty. Our cohort included younger men (age <65 years) which may explain the lower proportion of frailty than observed in other cohorts. Finally, our study focused on a European population and so the results should be extrapolated beyond this group with caution.

Our findings are consistent with the view that in addition to an increased susceptibility to falls among frail men, reduced bone strength contributes to susceptibility to fracture risk. Data from a recent trial among female nursing home residents suggest that treatment with long-acting bisphosphonate (zoledronic acid) is linked with an increase in bone density in this vulnerable group and provides therefore a real opportunity for prevention of fractures in this group based on targeting bone [30, 31].

In conclusion, a reduction in bone strength may in part explain the increased susceptibility to fracture among frail older people. Prevention of fractures in frail older people should include consideration of optimising bone health as well as preventing falls.

Key points

- Frailty is associated with reduced bone density and heel ultrasound parameters.
- Pre-frail men have ultrasound parameters intermediate between robust and frail men.
- Optimisation of bone health should be considered as a strategy to reduce fractures in frail older people.

## Supplementary data

Supplementary data mentioned in the text are available to subscribers in *Age and Ageing* online.

## Supplementary Material

Supplementary Data
