# Supplementary material for: Frailty and bone health in European men
Source: Age Ageing. 2016 Nov 16;46(4):635–41. doi: 10.1093/ageing/afw205 (PMC5859977; doi:10.1093/ageing/afw205)
Supplement: Supplementary Data [file afw205_aa-16-0377-file001.pdf]

Supplementary Table 1. Components of the Fried phenotype and bone health parameters

Component of the Fried frailty category (presence vs absence)[a]	Heel quantitative ultrasound			Areal bone mineral density	
	SOS	BUA	QUI	Lumbar spine	Femoral neck
	Adjusted β coefficient (95% CI)[b]			Adjusted β coefficient (95% CI)[a]	
Low physical activity	-4.6 (-8.6, -0.5)*	-0.9 (-3.1, 1.4)	-2.3 (-4.8, 0.2)	-0.033 (-0.084, 0.018)	-0.012 (-0.047, 0.024)
Exhaustion	-9.7 (-13.9, -5.5)***	-6.0 (-8.3, -3.6)***	-6.4 (-9.0, -3.8)***	-0.061 (-0.12, -0.01)	-0.047 (-0.085, -0.0097)*
Slow walking speed	-8.3 (-12.3, -4.3)***	-5.5 (-7.7, -3.2)***	-5.7 (-8.1, -3.2)***	0.026 (-0.016, 0.069)	-0.0095 (-0.039, 0.020)
Weakness	-4.9 (-9.6, -0.2)*	-2.3 (-4.9, 0.4)	-3.0 (-6.0, -0.09)*	0.025 (-0.029, 0.078)	-0.0098 (-0.047, 0.027)
Sarcopenia	-0.6 (-5.9, 4.7)	0.8 (-2.2, 3.7)	-0.004 (-3.3, 3.3)	-0.048 (-0.10, 0.0069)	-0.020 (-0.058, 0.018)

SOS: speed of sound (m/s), BUA: broadband ultrasound attenuation (dB/MHz), QUI: quantitative ultrasound index

Lumbar spine and femoral neck BMD is measured in g/cm2

[a]The number of subjects included in the analyses varies by component of the Fried frailty category from 3008 to 3211 for heel quantitative ultrasound

[b]Adjusted for age, BMI and centre

* p<0.05

** p<0.01

***p<0.001
